# Supplementary material for: Modeling glioblastoma heterogeneity as a dynamic network of cell states
Source: Mol Syst Biol. 2021 Sep 16;17(9):e10105. doi: 10.15252/msb.202010105 (PMC8444284; doi:10.15252/msb.202010105)
Supplement: Supplementary file 5 — Source Data for Figure 3 [file MSB-17-e10105-s001.zip › Figure3A_sourcedata/GSEA_3065/nosigdb_state3.GseaPreranked.1622718426028/OLSEN_ADRENERGIC.html]

Details for gene set OLSEN\_ADRENERGIC[GSEA]

|  || Dataset | state3 |
| Phenotype | NoPhenotypeAvailable |
| Upregulated in class | na\_neg |
| GeneSet | OLSEN\_ADRENERGIC |
| Enrichment Score (ES) | -0.65547544 |
| Normalized Enrichment Score (NES) | -2.196982 |
| Nominal p-value | 0.0 |
| FDR q-value | 0.0 |
| FWER p-Value | 0.0 |
Table: GSEA Results Summary

  

Fig 1: Enrichment plot: OLSEN\_ADRENERGIC      
 Profile of the Running ES Score & Positions of GeneSet Members on the Rank Ordered List

  

| PROBE | GENE SYMBOL | GENE\_TITLE | RANK IN GENE LIST | RANK METRIC SCORE | RUNNING ES | CORE ENRICHMENT || 1 | NCAM1 |  |  | 52 | 0.430 | 0.0230 | No |
| 2 | HSP90AA1 |  |  | 60 | 0.402 | 0.0494 | No |
| 3 | HSP90AB1 |  |  | 74 | 0.371 | 0.0729 | No |
| 4 | SOX11 |  |  | 117 | 0.325 | 0.0900 | No |
| 5 | LUC7L3 |  |  | 289 | 0.247 | 0.0870 | No |
| 6 | SLC38A1 |  |  | 459 | 0.207 | 0.0815 | No |
| 7 | SCG2 |  |  | 778 | 0.160 | 0.0557 | No |
| 8 | TUBB2B |  |  | 904 | 0.148 | 0.0514 | No |
| 9 | MAP1B |  |  | 975 | 0.140 | 0.0528 | No |
| 10 | CCT3 |  |  | 1198 | 0.122 | 0.0354 | No |
| 11 | KIF21A |  |  | 1855 | 0.084 | -0.0344 | No |
| 12 | KIF5C |  |  | 2183 | 0.069 | -0.0674 | No |
| 13 | MAPT |  |  | 3333 | 0.032 | -0.1974 | No |
| 14 | TUBB2A |  |  | 4022 | 0.015 | -0.2756 | No |
| 15 | ZFHX3 |  |  | 4494 | 0.001 | -0.3297 | No |
| 16 | ENO2 |  |  | 4676 | -0.003 | -0.3503 | No |
| 17 | HAND2 |  |  | 5195 | -0.016 | -0.4089 | No |
| 18 | HMGB2 |  |  | 5479 | -0.023 | -0.4399 | No |
| 19 | CRMP1 |  |  | 6243 | -0.048 | -0.5244 | No |
| 20 | TMSB15A |  |  | 6711 | -0.067 | -0.5737 | No |
| 21 | HDAC2 |  |  | 6905 | -0.076 | -0.5907 | No |
| 22 | CD24 |  |  | 7180 | -0.092 | -0.6160 | No |
| 23 | NREP |  |  | 7330 | -0.103 | -0.6263 | No |
| 24 | CCND1 |  |  | 7456 | -0.113 | -0.6330 | No |
| 25 | CDK6 |  |  | 7605 | -0.128 | -0.6414 | No |
| 26 | CKB |  |  | 7659 | -0.134 | -0.6384 | No |
| 27 | STMN1 |  |  | 7808 | -0.153 | -0.6452 | Yes |
| 28 | TUBB |  |  | 7813 | -0.154 | -0.6352 | Yes |
| 29 | OCIAD2 |  |  | 7906 | -0.167 | -0.6345 | Yes |
| 30 | H2AFZ |  |  | 7924 | -0.171 | -0.6249 | Yes |
| 31 | RAN |  |  | 7979 | -0.181 | -0.6189 | Yes |
| 32 | CBX1 |  |  | 7995 | -0.184 | -0.6082 | Yes |
| 33 | ATP6V0E2 |  |  | 8025 | -0.189 | -0.5988 | Yes |
| 34 | HIST1H4C |  |  | 8075 | -0.198 | -0.5910 | Yes |
| 35 | RBP1 |  |  | 8081 | -0.200 | -0.5781 | Yes |
| 36 | SOX4 |  |  | 8123 | -0.209 | -0.5687 | Yes |
| 37 | FSCN1 |  |  | 8144 | -0.214 | -0.5565 | Yes |
| 38 | JPT1 |  |  | 8212 | -0.231 | -0.5486 | Yes |
| 39 | CALM2 |  |  | 8295 | -0.257 | -0.5407 | Yes |
| 40 | UCHL1 |  |  | 8299 | -0.258 | -0.5237 | Yes |
| 41 | PAFAH1B3 |  |  | 8326 | -0.264 | -0.5088 | Yes |
| 42 | YWHAQ |  |  | 8360 | -0.278 | -0.4939 | Yes |
| 43 | H3F3A |  |  | 8442 | -0.311 | -0.4822 | Yes |
| 44 | BEX1 |  |  | 8450 | -0.314 | -0.4617 | Yes |
| 45 | ZNF428 |  |  | 8462 | -0.322 | -0.4413 | Yes |
| 46 | TAGLN3 |  |  | 8479 | -0.330 | -0.4209 | Yes |
| 47 | RPS7 |  |  | 8486 | -0.333 | -0.3991 | Yes |
| 48 | MLLT11 |  |  | 8489 | -0.335 | -0.3767 | Yes |
| 49 | RPL38 |  |  | 8496 | -0.339 | -0.3545 | Yes |
| 50 | TUBA1A |  |  | 8499 | -0.341 | -0.3318 | Yes |
| 51 | PTMA |  |  | 8523 | -0.348 | -0.3109 | Yes |
| 52 | CHCHD2 |  |  | 8525 | -0.348 | -0.2875 | Yes |
| 53 | SUMO2 |  |  | 8573 | -0.383 | -0.2671 | Yes |
| 54 | GAPDH |  |  | 8615 | -0.418 | -0.2436 | Yes |
| 55 | PPIA |  |  | 8626 | -0.432 | -0.2156 | Yes |
| 56 | GAP43 |  |  | 8637 | -0.441 | -0.1869 | Yes |
| 57 | RPL27 |  |  | 8639 | -0.441 | -0.1573 | Yes |
| 58 | HMGN2 |  |  | 8644 | -0.449 | -0.1274 | Yes |
| 59 | MZT2B |  |  | 8681 | -0.495 | -0.0982 | Yes |
| 60 | H3F3B |  |  | 8687 | -0.504 | -0.0648 | Yes |
| 61 | FABP5 |  |  | 8699 | -0.518 | -0.0311 | Yes |
| 62 | HINT1 |  |  | 8712 | -0.547 | 0.0045 | Yes |
Table: GSEA details [plain text format]

  

Fig 2: OLSEN\_ADRENERGIC: Random ES distribution      
 Gene set null distribution of ES for **OLSEN\_ADRENERGIC**
